# Frequency of Food Consumption and Self-reported Diabetes among Adult Men and Women in India: A Large Scale Nationally Representative Cross-sectional Study

**DOI:** 10.4172/2155-6156.1000474

**Published:** 2015-01-02

**Authors:** Sutapa Agrawal

**Affiliations:** South Asia Network for Chronic Disease, Public Health Foundation of India, New Delhi NCR, India

**Keywords:** Fruit, Pulses and beans, Milk or curd, Fish, Self-reported diabetes, Men, Women, NFHS-3, India

## Abstract

**Background:**

Recent studies have shown that the choice of foods and frequency of intake plays a role in diabetes prevention. We examined the association between frequency of consumption of specific food items and the occurrence of diabetes in adult Indian population.

**Methods:**

Cross sectional data of 99,574 women and 61,361 men aged 20-49 years who participated in India’s third National Family Health Survey conducted during 2005-06 was used for this study. Association between frequency of food intake such as daily, weekly, occasionally and never, and prevalence of diabetes were estimated using multivariable logistic regression models after adjusting for body mass index, tobacco smoking, alcohol drinking, television watching and socio-economic and demographic characteristics, stratified by sex.

**Results:**

In men, weekly (OR:0.64; 95%CI:0.47-0.88) and occasional (OR:0.60; 95%CI:0.44-0.81) consumption of milk/curd, weekly (OR:0.48; 95%CI:0.27-0.87) and occasional (OR:0.52; 95%CI:0.28-0.99) consumption of pulses/beans and consumption of fruits (OR ranges from 0.33 to 0.39) was associated with a significantly lower likelihood of diabetes whereas daily (OR:0.55; 95%CI:0.34-0.88) or weekly (OR:0.56; 95%CI:0.35-0.90) pulses/beans consumption and fruits intake (OR ranges from 0.36 to 0.46) was associated with a lower likelihood of diabetes in women.

**Conclusion:**

This study has confirmed findings from high income countries that diabetes among adult Indians, which is large and increasing, might be contained by regular consumption of vegetarian foods including pulses, beans, fruits and dairy products. However, this is an observational finding and uncontrolled confounding cannot be excluded as an explanation for the association. More epidemiological research with better measures of food intake and clinical measures of diabetes is needed in a developing country setting to validate the findings.

## Introduction

India is experiencing an alarming increase in the incidence and prevalence of type 2 diabetes mellitus [1–6] both in rural [7,8] and urban areas [9–12], with higher prevalence in south than in north India [13]. The increasing health challenge of diabetes in Asia as well as in India has been well established in a series of recent studies [4,6,14–18]. Although obesity is the most important risk factor for type 2 diabetes [19], lifestyle intervention trials that include dietary changes have been shown to be effective in preventing the development of diabetes [20]. Recent evidences have emerged from developed countries that certain foods and dietary factors may be associated with diabetes [21] and thus the choice of foods may play a role in diabetes prevention.

Studies showed that a prudent diet is a key component of a healthy lifestyle for preventing type 2 diabetes [8,22]. While fish, particularly oily fish, is generally considered to be an important part of a healthy diet and lowers the risk of diabetes [23–26] concerns have been raised that fish consumption especially shellfish [25], is also associated with a higher risk of developing diabetes [5,27,28]. Diets high in animal protein are also associated with an increased diabetes risk [29]. Evidences from Asian diet found that diets high in legumes [13,30,31] and soy [30,32,33] may be beneficial in preventing diabetes whereas, a diet low in whole grains and higher in processed meats appears to increase the risk [21,24]. Consumption of fruit and vegetables [34] has shown inverse associations with the risk of diabetes. A World Health Organization expert consultation recommended a minimum intake of 400 g or five portions (based on an average portion weighing 80 g) of combined fruits and vegetables per day for the prevention of several major non communicable diseases, including diabetes (WHO, 2003). Other dietary factors that have been related to reduced risk of type 2 diabetes include dairy products [35]. Some studies focusing on a dietary pattern approach have found some definite dietary patterns to be associated with the incidence of diabetes [22,36] or biomarkers of diabetes development [37].

Given the high growing prevalence of diabetes in India [6], the role of various food items needs to be examined in relation to its prevalence. At present, there is a dearth of empirical research in India regarding the role of different food items in the prevention of diabetes. In order to develop effective dietary public health strategies for diabetes prevention, it would be useful to examine the association of various food consumption with risk of diabetes in Indians. India’s third National Family Health Survey (NFHS-3, 2005-06) collected data from 109,041 households on a wide range of dietary, societal, lifestyle, and environmental determinants of morbidity and chronic ailments, including diabetes, for adult men aged 15-54 years and women aged 15-49 years (IIPS and Macro International, 2007), and covered regions comprising more than 99% of India’s population, provide a unique opportunity to study the association between various types of food consumption and the risk of diabetes in a large nationally representative sample of adult men and women in India.

## Materials and Methods

### Data

Data from India’s third National Family Health Survey (NFHS-3, 2005-06) were used for this study. Details of survey objective, survey method including sampling frame and questionnaire used are provided elsewhere (IIPS and Macro International, 2007; www.nfhsindia.org). Briefly, this survey was designed on the lines of the Demographic and Health Surveys (DHS) (available at www.measuredhs.com) that have been conducted in many developing countries since the 1980s. NFHS-3 collected demographic, socioeconomic, and health information from a nationally representative probability sample of 124,385 women aged 15-49 years and 74,369 men aged 15-54 years residing in 109,041 households. The sample is a multi-stage cluster random sample with an overall response rate of 98%. The samples were geo-coded to the primary sampling unit, district, and state to which they belonged. The data was obtained from face-to-face interviews conducted in the respondents’ homes. All states of India are represented in the sample (except the Union Territories), covering more than 99% of country’s population. The survey was conducted using an interviewer-administered standardized questionnaire in the native language of the respondent and a total of 18 languages were used in the survey with back translation into English to ensure accuracy and comparability. The analysis presented in this study is restricted to the 99,574 women and 56,742 men aged 20-49 years living in the sample households, to ensure comparability and to avoid any cases of childhood diabetes.

### Response variable

The survey asked participants the question, *‘Do you currently have diabetes?’*. Neither data on physician reported diagnosis of diabetes or fasting blood glucose was available in the NFHS-3 to verify a self-reported diagnosis. In our analysis, reported prevalence of diabetes is the outcome of interest.

### Dietary predictor variables and covariates

The survey collected information on demographic, socioeconomic factors, anthropometric measurements and dietary intake. Consumption of selected foods was assessed by asking, *‘How often do you yourself consume the following items: daily, weekly, occasionally or never?’* related to fish consumption, milk or curd, pulses and beans, green leafy vegetables, other vegetables, fruits, eggs, chicken or meat. Frequency of watching television (almost every day, at least once weekly, less than once weekly, not at all) was used as a measure of sedentary behaviour. The information exposure to tobacco smoke was–yes–active smoking (person currently smokes) and no smoking (the person has never smoked). Use of alcohol was quantified as drinks taken almost every day, about once weekly, less than once weekly and never. Respondents were weighed using a solar powered scale with an accuracy of ± 100 g. Their height was measured using an adjustable wooden measuring board, specifically designed to provide accurate measurements (to the nearest 0.1 cm). Indian adult population standard (Indian Consensus Group, 1996; WHO expert consultation, 2004; Mishra et al., 2009) categories of Body Mass Index (BMI, kg/m^2^) were used: ≤ 18.5 kg/m^2^ (underweight); 18.5 to 22.9 kg/m^2^ (normal), 23.0 to 24.9 kg/m^2^ (overweight), and ≥ 25.0 kg/m^2^ (obese). Other covariates in our analysis include: age (20-29, 30-39, 40-49 years); education (illiterate, literate but less than middle school complete, middle school complete but less than high school complete, high school complete or higher); religion (Hindu, Muslim, Christian, Sikh, Others); caste/tribe status (scheduled caste, scheduled tribe, other backward class, others, missing caste); wealth status (based on 33 assets and housing characteristics graded lowest, second, middle, fourth, highest); and place of residence (urban, rural). For a detailed definition of some variables (Table 1).

### Statistical analysis

Descriptive statistics were calculated with use of standard methods (such as frequencies and percentages) in men and women separately. Prevalence of diabetes was computed as percentage prevalence. Differences were tested using χ^2^ tests. Multiple logistic regression models were used to estimate the odds ratios of daily and weekly consumption of various food items on risk of diabetes after controlling for potential confounders. The following models were constructed to account for potential confounders and mediators: Model 1 presents unadjusted results; Model 2 presents results adjusted for BMI, lifestyle factors and socio-demographic factors which may be confounders to exhibit any independent effect of food consumption on diabetes prevalence. As certain states and certain categories of respondents were oversampled, in all analyses sample weights were used to restore the representativeness of the sample (IIPS and Macro International, 2007).

As the effects of various food intakes on the prevalence of diabetes are likely to vary by sex, due to the large gender differences in nutritional status in India, the susceptibility to disease, and access to treatment and care in a developing country in general, the analysis was carried out separately for women and men. Results are presented in the form of odds ratios (ORs) with 95 percent confidence intervals (95%CI). All the analysis including the logistic regression models were conducted using the SPSS statistical software package, version 19 (IBM SPSS Statistics, Chicago, IL, USA).

### Ethics statement

The analysis is based on secondary analysis of existing survey data with all identifying information removed. The NFHS-3 survey was approved by the International Institute for Population Sciences ethical review board and the Indian government. Participation in the survey was totally voluntary. The survey obtained written informed consent from each respondent (in this case, men and women included in the analysis) before asking questions, and separately before obtaining height and weight.

## Results

### Characteristics of the study population and prevalence of diabetes

**Figure 1:**
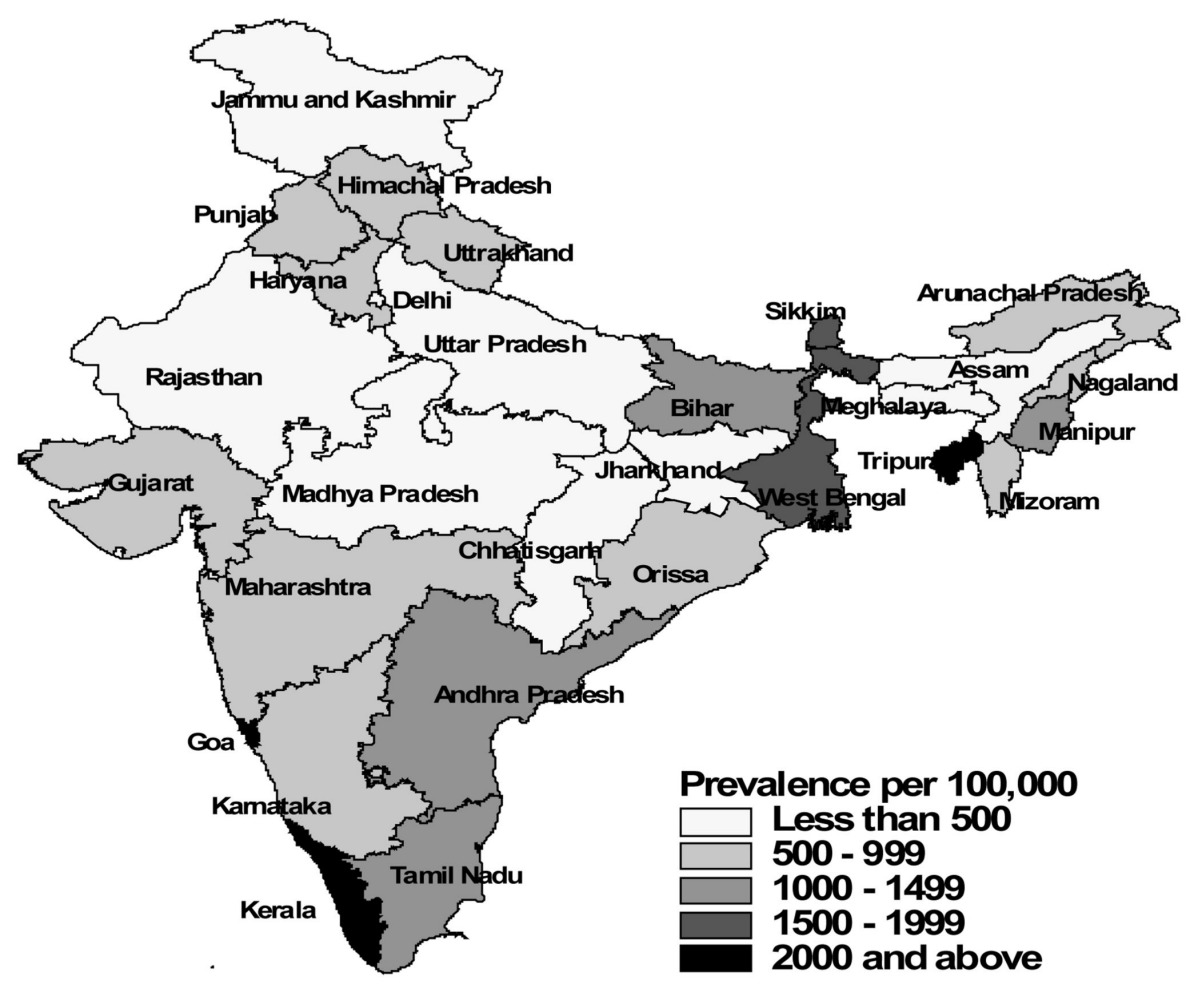
Diabetes – Rural India.

**Figure 2:**
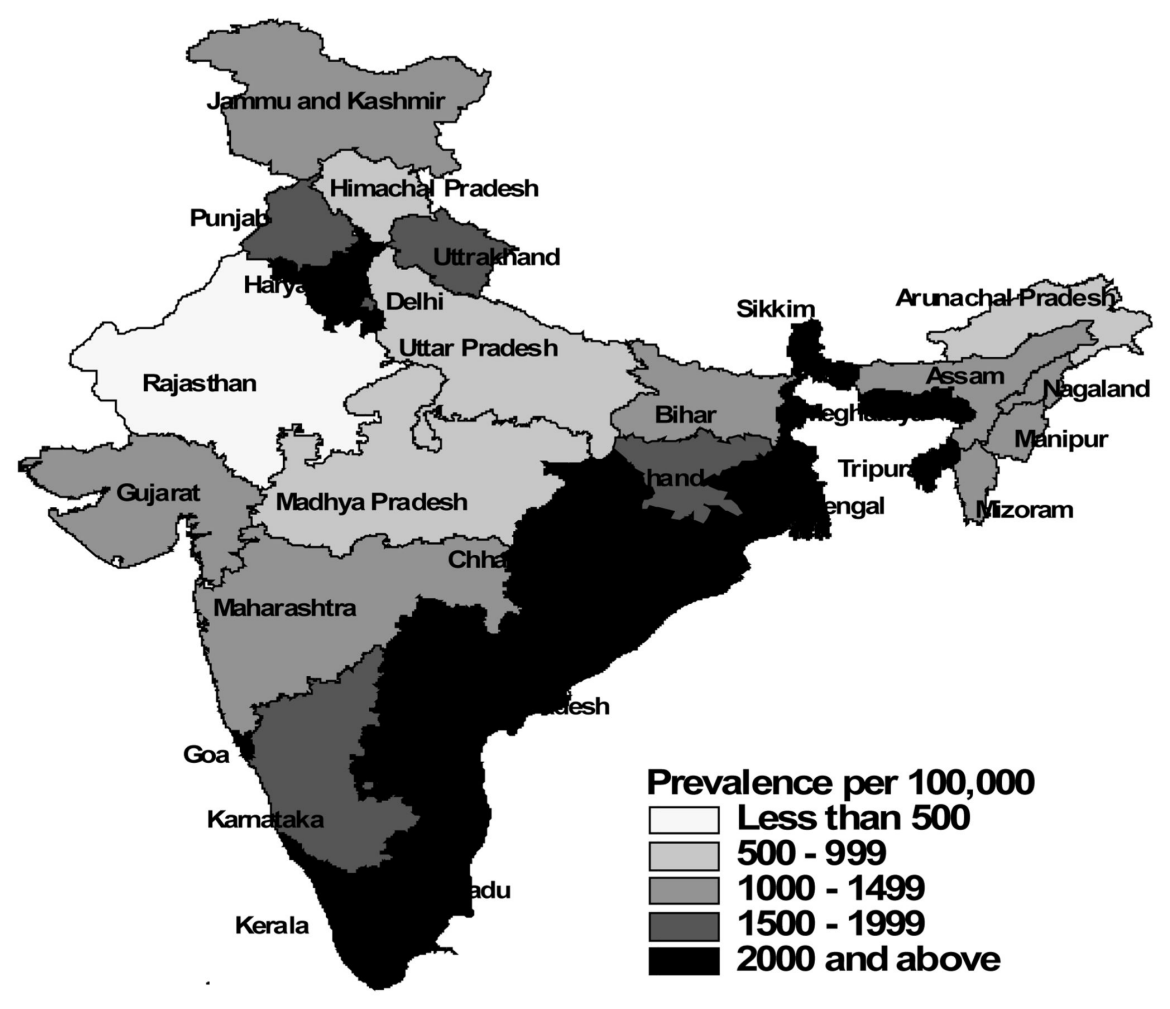
Diabetes – Urban India.

**Figure 3:**
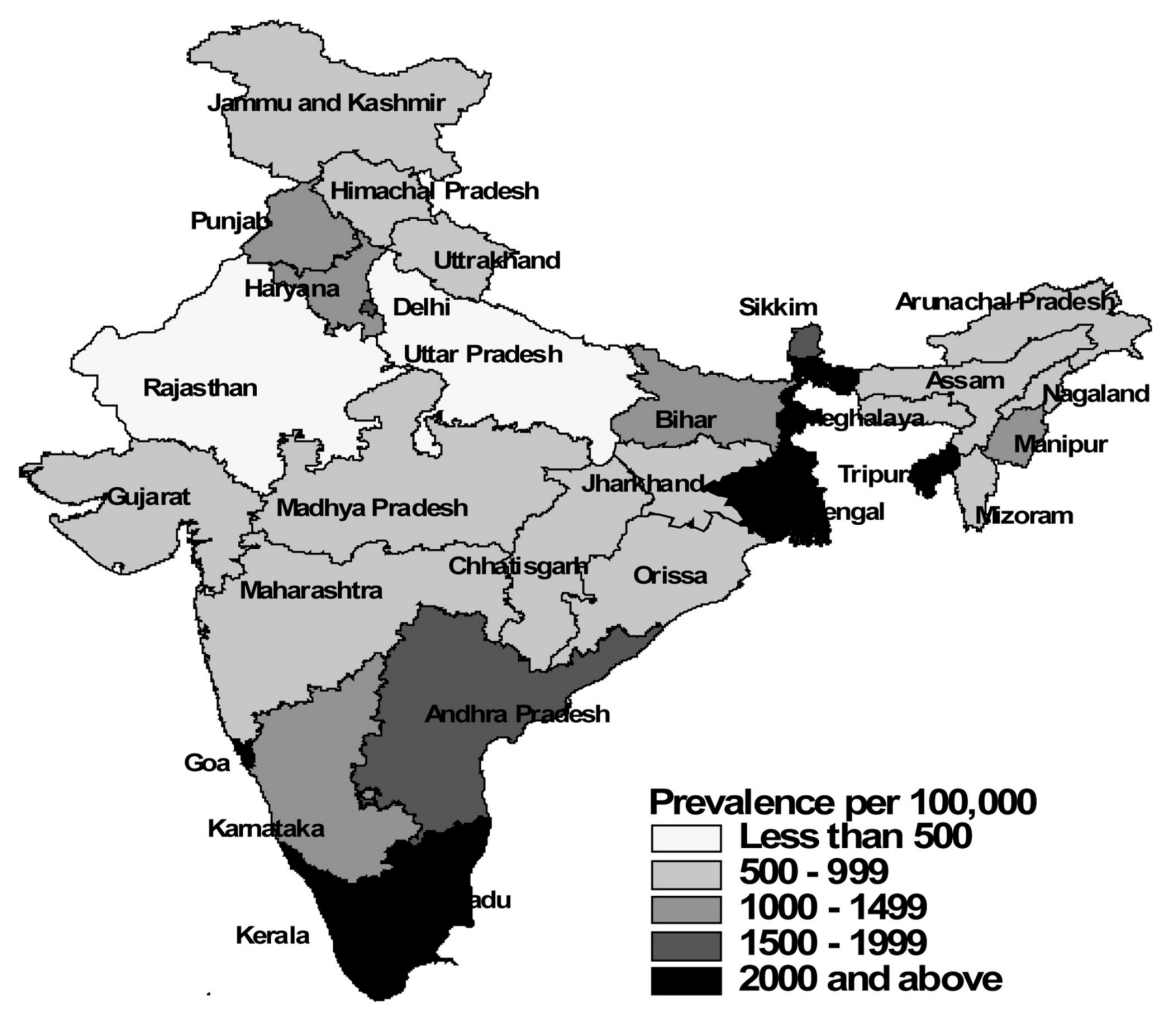
Diabetes – India Total.

**Figure 4:**
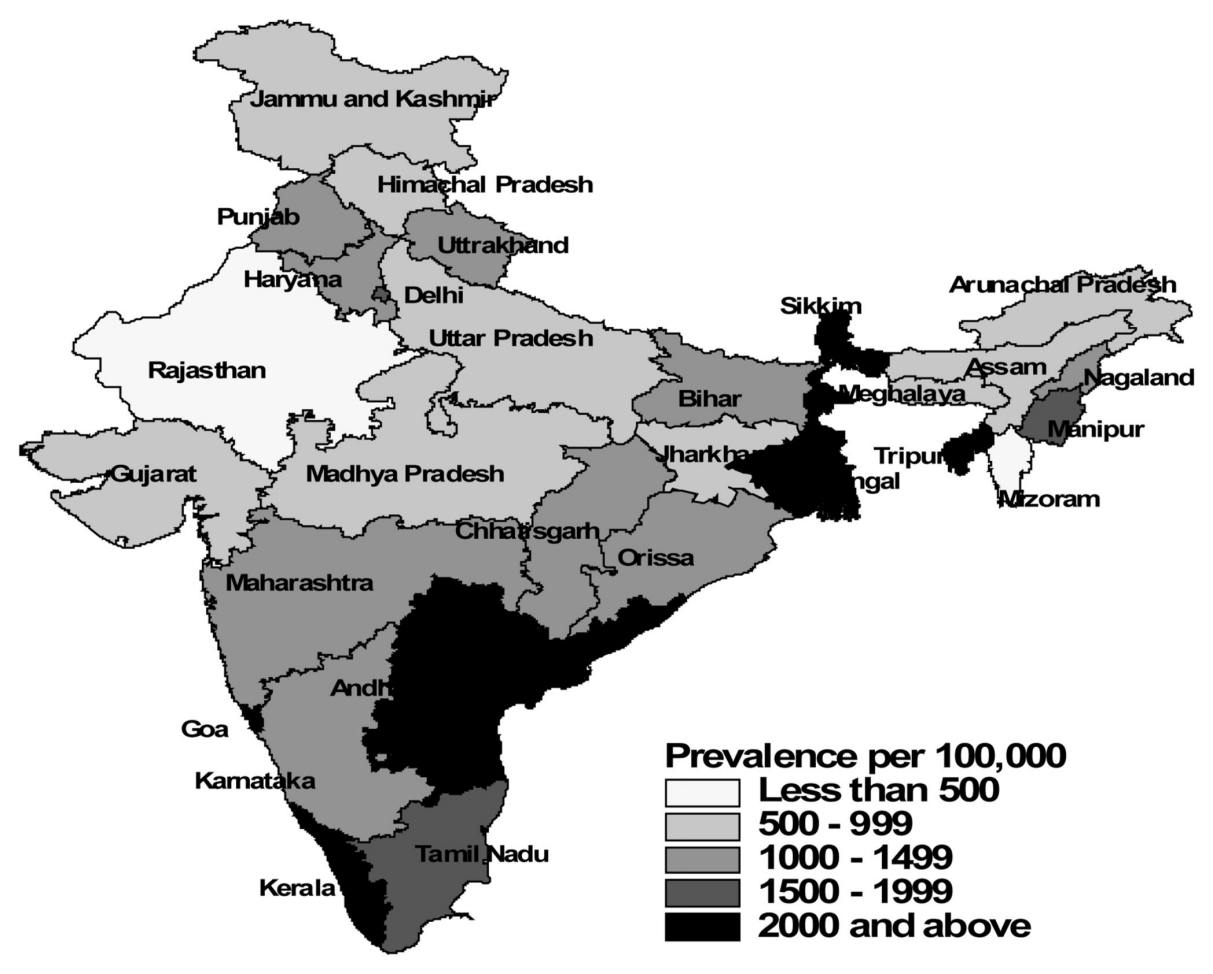
Diabetes – Men.

**Figure 5:**
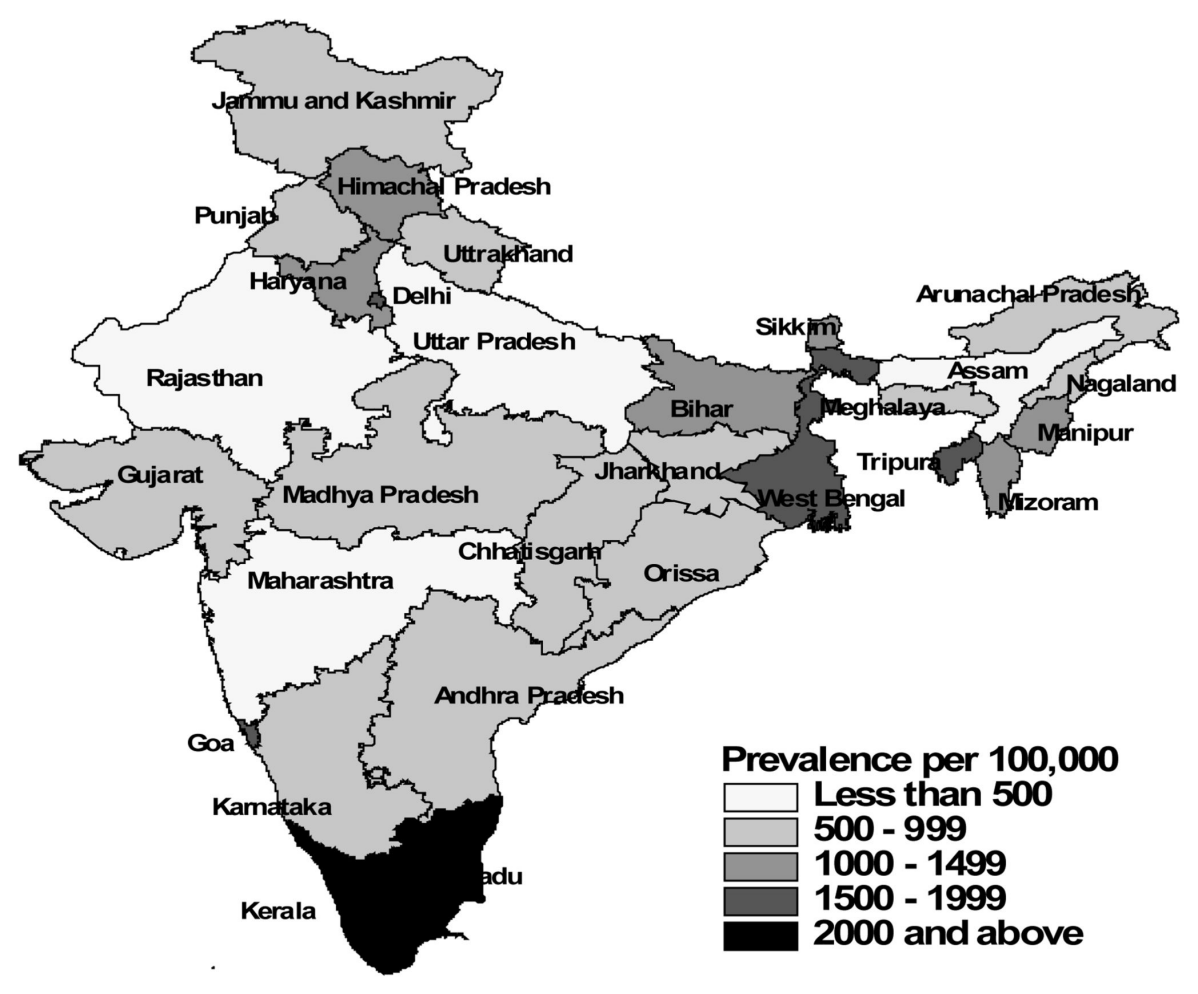
Diabetes – Women.

Table 1 shows the characteristics of the study population separately for men and women, according to their food consumption, selected risk factors and socio-economic and demographic characteristics, and the corresponding prevalence of diabetes among them (Figures 1-5). The overall prevalence of diabetes was higher among men (1.3%) than among women (1.1%). Diabetes was more common among both men and women who never consumed milk or curd, fruits or vegetables, consumed eggs, fish, chicken or meat daily or weekly, never consumed fruits, who were either overweight or obese, who watched television almost every day, and in those who were the oldest age group, lived in urban areas and in wealthier households (all p<0.0001). Daily or weekly pulses and beans consumption was associated with a lower prevalence of diabetes among men (1.5%) and women (1.0%) than observed in people never eating pulses and beans (men 2.6% and women 2.3%). Significant associations between age and diabetes prevalence were observed. Diabetes prevalence increased according to the wealth of the household and was almost double in urban women and men compared with their rural counterparts. No differences in prevalence of diabetes were seen for smoking tobacco or alcohol consumption or by educational attainment.

### Association between frequency of consumption of specific food items and diabetes prevalence among men

Unadjusted odds (Model 1, Table 2) of suffering from diabetes are lower among men who consume pulses and beans daily (OR:0.57;95%CI:0.32-0.99), weekly or occasionally, fruits daily (OR:0.59; 95%CI:0.42-0.83), weekly (OR:0.45; 95%CI:0.33-0.63) or occasionally (OR:0.36; 95%CI:0.26-0.49); milk weekly (OR:0.56; 95%CI:0.42-0.75) or occasionally; higher for those who consumed eggs daily (OR:1.74; 95%CI:1.28-2.38) or weekly, fish daily (OR:2.48; 95%CI:1.92-3.22) or weekly and chicken or meat weekly (OR:1.60; 95%CI:1.31-1.95) as compared to those who never consumed them. When the BMI, lifestyle factors, socio-economic control variables and other covariates are included in Model 2 (Table 2), weekly (OR:0.64; 95%CI:0.47-0.88) and occasional (OR:0.60; 95%CI:0.44-0.81) consumption of milk or curd, weekly (OR:0.48; 95%CI:0.27-0.87) and occasional (OR:0.52; 95%CI:0.28-0.99) consumption of pulses and beans, consumption of fruits (OR ranges from 0.33 to 0.39), daily chicken or meat intake (OR:0.31; 95%CI:0.12-0.82) was still associated with a significantly reduced risk of diabetes whereas daily (OR:2.46; 95%CI:1.66-3.65) and weekly (OR:1.77; 95%CI:1.24-2.53) fish consumption was associated with a higher diabetes risk in men.

Considering the BMI status, diabetes was 1.8 times higher among obese (OR:1.78; 95%CI:1.44-2.20) and 1.6 times higher among overweight men (OR:1.56; 95%CI:1.25-1.94) in the adjusted analysis. With other variables controlled, age has a positive and statistically significant effect on diabetes among men. The odds of suffering from diabetes were seven times higher (OR:7.19; 95%CI:5.65-9.16) among men aged more than 40 years. Educated men had lower odds of diabetes (OR ranges from 0.73 to 0.78). Caste/tribe status is also significantly associated with lower odds of diabetes in men. By contrast, highest wealth index remained significantly associated with increased (OR:2.98; 95%CI:2.01-3.41) risk of diabetes in men in the adjusted analysis. However, no effect of green leafy vegetables, eggs, chicken or meat consumption, tobacco smoking, alcohol consumption, TV watching, religion and place of residence on diabetes was found in the adjusted analyses among men.

### Association between frequency of consumption of specific food items and diabetes prevalence among women

Unadjusted odds (Model 1, Table 3) of suffering from diabetes were significantly lower lower among those who consumed pulses and beans daily (OR:0.43; 95%CI:0.27-0.67), weekly (OR:0.41; 95%CI:0.26-0.65) or even occasionally(OR:0.57; 95%CI:0.36-0.92); consume fruits weekly or occasionally; eggs occasionally; higher for those who consumed eggs daily (OR:1.76; 95%CI:1.34-2.33) or weekly (OR:1.29; 95%CI:1.11-1.50), fish daily (OR:2.58; 95%CI:2.13-3.14) or weekly (OR:1.54; 95%CI:1.32-1.80), and chicken or meat weekly (OR:1.44; 95%CI:1.23-1.69) compared to those who never consumed legumes. Even when the BMI lifestyle factors and socio-economic control variables are included in Model 2 (Table 3), effect of daily (OR:0.51; 95%CI:0.32-0.81) or weekly (OR:0.51; 95%CI:0.32-0.81) pulses and beans consumption still has a reduced and statistically significant effect on the prevalence of diabetes among women; among other diets, frequency of consumption of fruits (OR ranges from 0.36 to 0.46) was associated with a significantly reduced risk of diabetes in women whereas daily (OR:1.72; 95%CI:1.26-2.33) and weekly (OR:1.41; 95%CI:1.07-1.87) fish consumption was associated with a higher prevalence of diabetes risk.

Considering BMI status, the prevalence of diabetes was 2.4 times higher among obese (OR:2.37; 95%CI:2.01-2.79) and 1.6 times higher among overweight women (OR:1.59; 95%CI:1.30-1.94) in the adjusted analysis. With other variables controlled, age has a positive and statistically significant effect on diabetes among women. The odds of suffering from diabetes were eight times higher (OR:8.03; 95%CI:6.49-9.93) among women aged more than 40 years. Literate women with either <middle school (OR:1.38; 95%CI:1.14-1.68) or middle school completed education (OR:1.49; 95%CI:1.24-1.79) also had higher odds of diabetes prevalence. Urban women (OR:1.44; 95%CI:1.24-1.68) and women belonging to the Christian religion have significantly higher odds (OR:1.51; 95%CI:1.12-2.03) of diabetes. The wealth index also remained significantly associated with increased risk of diabetes (ORs ranges from 1.41 to 1.93) in women in the adjusted analysis. However, no effect was found on diabetes from the consumption of milk or curd, green leafy vegetables, eggs, chicken or meat, tobacco smoking, alcohol consumption, TV watching and caste or tribe status in the adjusted analyses among women.

## Discussion

In this large nationally representative sample of adult men and women in India, significant positive associations between daily and weekly fish intake and diabetes were observed whereas consumption of fruit, pulses and beans, milk and curd were inversely associated with risk of diabetes. These associations are robust after controlling for other risk factors such as, BMI, tobacco smoking, alcohol drinking, and a range of socio-economic and demographic characteristics of the population.

Our study is the cross sectional, population-based study to look at frequency of food consumption and prevalence of diabetes in India, and adds to the limited data on the associations between food intake and diabetes prevalence in developing countries. Our results are in line with the results of previous epidemiological studies which have shown inverse associations between the consumption of fruit and vegetables and the risk of diabetes or 2 h post-load glucose concentrations [34,38]. Although epidemiologic studies in the West, where the average daily intake (in grams) is much lower than in India, have yielded inconsistent associations on pulses and beans consumption and chronic conditions [30,33,39–41], consumption of legumes is recommended by the European [42], Canadian [43] and American Diabetes Associations [44–46] as a means of increasing one’s daily fiber intake and lowering glycemic index (GI) for diabetes control. Studies focusing on legumes specifically, also showed inverse associations with diabetes in some of the studies [47,48] and thus evaluations of dietary patterns have identified legumes as an important component of both the ‘prudent diet’ [37] and ‘Mediterranean diet’ [22], which have been associated with a lower risk of diabetes in some studies [47,48], including those from developing countries [31]. The protective effect of legumes on diabetes may be due to multiple biological reasons, including increased fiber content in the diet [49], a reduction in the GI of mixed meals [50], or both. In addition, legumes contain polyphenols, such as isoflavones and lignans, which have an antioxidant effect and may be responsible for the protective role of legumes against the development of diabetes [51].

A cross sectional study of Seventh-Day Adventists in California showed a lower risk of diabetes among vegetarians, who consumed more legumes, fruits, and nuts in the absence of meat intake [52,53]. Fruit and berries and vegetables are rich sources of antioxidant compounds such as carotenoids, vitamin C, vitamin E and flavonoids, and of fiber [22] and also contain other potential compounds, such as phytates or isoflavones, which may have additive or synergistic effects [54] and may have a protective effect against development of diabetes by relieving oxidative stress that interferes with the glucose uptake by cells [55,56]. Fiber specifically derived from vegetables or fruit has, however, not been found associated with diabetes risk [57].

Several studies have suggested that dairy products may have favorable effects on body weight, the major determinant of type 2 diabetes [3,58–60]. An inverse cross-sectional association between dairy intake and insulin resistance syndrome (IRS) was observed in men [61]. Another study [35] found that dietary patterns characterized by increased dairy consumption may reduce risk of type 2 diabetes (T2DM). A number of observational studies mostly conducted in west, showed a consistent inverse association between dairy intake and the prevalence of IRS and type 2 diabetes mellitus [36,62–67] with the exception of a recent study of middle-aged Chinese women [68]. Results from a meta-analysis of observational studies showed that the odds for incident T2DM was 0.86 (95% CI: 0.78–0.93) for the highest vs the lowest dairy intakes (3–5 vs <1.5 servings/d) [69]. A recent meta-analysis of cohort studies found an inverse association of daily intake of dairy products, especially low-fat dairy, with T2DM, indicating a beneficial effect of dairy consumption in the prevention of T2DM development [46]. The CARDIA study showed an inverse association between intake of dairy products and development of insulin resistance in young adults [35]. In the present study, intake of dairy products at least weekly suggested an inverse and significant association in men but non-significant inverse association was observed in case of women, is in accordance with the finding by [35]. The Diabetes India website (www.diabetesindia.com) does recommend consuming up to 1 litre of milk daily as part of a diabetic diet. Factors that have been shown to impact the strength of the associations include: the amount and type of dairy products, their fat levels and nutrient constituents [70]. Electrolytes in dairy foods, such as calcium and magnesium, may lower the risk of type 2 diabetes [35]. Other major components in dairy products, such as lactose and dairy protein, may enhance satiety and reduce the risk of overweight and obesity, a the major risk factor [35] since obesity does not contribute as much as age, for example, in this study.

The emerging scientific evidence for fish and diabetes association is not consistent and findings from cross-sectional studies worldwide have reported inverse [24,26], no [71], or positive [28] associations between habitual/daily fish intake and diabetes. Our finding of daily and weekly fish consumption increasing the risk of diabetes was robust, suggesting that a non-vegetarian diet is harmful. However, this finding warrants further investigation looking into the cooking methods and mechanisms, which vary throughout the country. The method of preparation of fish (frying and the type and amount of cooking fat used) and the accompanying condiments with which fish is often served in India may not be beneficial for diabetes rather than the fish itself. Frying fish, especially deep frying, might produce transfatty acids, which might modify the beneficial effect of fish. The effect of fish intake on glucose metabolism may differ according to cooking method. Salting and drying, which are used to preserve fish, can also modify the association between fish intake and risk of diabetes. Greater shellfish intake has been found to be associated with increased risk of diabetes [25] and the coastal states of India where plentiful sea/salt water/shellfish are available are also the states where diabetes prevalence is higher [5].

The strength and limitations of this investigation also merit consideration. The strengths of our study include the use of large nationally representative study sample which allows comparisons to be made between men and women and the ability to examine this association in adult Indian population. Also rigorous efforts were made in the NFHS-3 to obtain reliable self-reported data: the survey used local terminology and commonly understood terms to describe the disease, rigorously trained interviewers, supervisors and standard quality checks (IIPS and Macro International, 2007).

The study has some limitations. This is an observational finding and uncontrolled confounding cannot be excluded as an explanation for the association. The misclassification of dietary information in NFHS-3 data, although unavoidable, would most likely not allow for true associations. Also, there is a possibility that the information derived from the NFHS-3 questionnaire, while critical to measure true dietary intake, are self-reported and thus may not meet the standards of validity despite the fact that NFHS-3 is a part of the Demographic and Health Surveys (available at www.measuredhs.com) conducted in more than 80 countries since 1980s [5].

Our study outcome was defined on the basis of self-reported diabetes, although interviews were conducted in person using a standardized instrument. Understandably, the prevalence of self-reported diabetes in our study was lower (about 1%) than prevalence estimates derived using bio-medical diagnostics diabetes in studies conducted in different parts of the country [1,4,7–11] but those were not nationally representative and conducted in some pockets/regions of India. Our study, a population based, nationally representative and focusing on young people (<60 years) in whom diabetes is less common [28], have shown lower prevalence of diabetes in our population which is because studies in India have demonstrated that many people with diabetes remain undiagnosed (www.diabetesfoundationindia.org). Individuals with undetected diabetes may have been misclassified as nondiabetic individuals, resulting in attenuated associations. The prevalence of undiagnosed diabetes in India is higher than diagnosed diabetes; thus, more people remain undiagnosed than those who self report diabetes [72,73]. Self-reported data, especially in rural areas, can be flawed owing to several factors such as lack of awareness, low educational status, limited access to health services and hesitation to disclose diagnosed diseases [28] but previous research has shown good agreement for self-reported diabetes when compared with medical records in a US population [74] and that self-reported health conditions demonstrate the expected relationship with socioeconomic status in India [75]. In addition, our analyses considering respondents who reported ‘unknown’ for diabetes status were nearly identical to the main analyses (data not shown). Although our sample was relatively young (<50 years for women and men both), it is representative of the young population of profile of India; 84% of the Indian adult population (18–69 years) and 47% of the total Indian population at all ages fall within the ages covered by this study (Registrar General of India, 2001). Our study does exclude approximately 14% of the Indian population (men and women over the age of 50) due to the sample design of the NFHS. The prevalence of diabetes increases with age and whether a similar SES–diabetes relationship exists among middle and older age groups in all parts India is not clear [76], although our findings are consistent with the previous studies that have included older ages.

We were also unable to distinguish between Type 1 and 2 diabetes diagnoses as there was no clinical confirmation of the reported cases. Under and over reporting could lead to a biased estimation of the association between dietary factors and diabetes [5]. Although we adjusted for several confounding variables, we cannot exclude the possibility of residual confounding. Also, given the high proportion of undiagnosed diabetes in developing countries (www.worlddiabetesfoundation.org) where less than half of people with diabetes are diagnosed, there is a possibility that the exposure was associated with the likelihood of testing for diabetes, which may result in detection bias. Since this is a cross sectional study, the entire study was with known diabetic subjects who might have altered their diet due to dietary advice based on diabetes control and on the complications of diabetes. Therefore the dietary choices of self-reported diabetic subjects might have been modified to manage diabetes.

Valid data on physical activity was not available in NFHS-3 which is a limitation of this study since persons with healthier diets may be physically more active than other persons [25], the lack of physical activity data in particular may have confounded the results. It is, however possible that, physical activity has in part been accounted for indirectly by adjusting for body mass index. In the present study, adjustment for socioeconomic and demographic factors, residential location, religion and caste/tribe status of the respondents did not markedly modify the adjusted result, suggesting that the associations found are not completely explained by non-dietary lifestyle factors. Further studies are needed to determine whether the association between diet and diabetes is mediated by assumed nutrients or by lifestyle and socioeconomic and demographic factors related to frequency of food consumption [76–86].

In conclusion, the results of the present study suggest an inverse association between frequency of intakes of fruits, pulses and beans, milk and curd and risk of diabetes while positive association was observed with daily and weekly fish intake and occurrence of diabetes in Indian adult population. Although the overall prevalence of diabetes was actually very low among the participants of the study, these results nevertheless adds to the no or limited evidence in developing countries that shows the beneficial effect of consuming vegetarian diet in countering the development of diabetes. These findings support current public health recommendations encouraging consumption of fruits and vegetables as part of a balanced diet and place particular emphasis on the important and independent role that both frequency and variety in vegetarian diet intake may play in helping to prevent the development of diabetes. These findings need further validation by longitudinal and clinical studies but may well have public health significance for the Indian population. More epidemiological research with better measures of frequency of food intake and clinical measures of diabetes are needed to validate the findings in a developing country.

## Figures and Tables

**Table 1:** Sample distribution and prevalence of diabetes (%) among men **(n=56,742)** and women **(n=99,574)** aged 20-49 according to frequency of consumption of specific food items and other selected risk factors and background characteristics, India 2005-06.

Variables	Men			Women		
	Total N (%)	Diabetes N (%)	Χ^2^p value	Total N (%)	Diabetes N (%)	Χ^2^p value
Milk or curd			<0.0001			<0.0001
Daily	26307(46.4)	391(1.5)		40366(40.5)	492(1.2)	
Weekly	11554(20.4)	117(1.0)		15071(15.1)	138(0.9)	
Occasionally	14757(26.0)	138(0.9)		32918(33.1)	302(0.9)	
Never	4114(7.3)	74(1.8)		11202(11.3)	117(1.0)	
Pulses and beans			<0.0001			<0.0001
Daily	29863(52.6)	437(1.5)		52440(52.7)	538(1.0)	
Weekly	21705(38.3)	219(1.0)		36597(36.8)	360(1.0)	
Occasionally	4660(8.2)	51(1.1)		9663(9.7)	131(1.4)	
Never	505(0.9)	13(2.6)		852(0.9)	20(2.3)	
Green leafy vegetables			0.149			0.090
Daily	33982(59.9)	453(1.3)		64095(64.4)	674(1.1)	
Weekly	19270(34.0)	231(1.2)		28606(28.7)	286(1.0)	
Never/Occasionally	3480(6.1)	35(1.0)		6840(6.9)	89(1.3)	
Fruits			<0.0001			<0.0001
Daily	7320(12.9)	125(1.7)		12789(12.9)	206(1.6)	
Weekly	19368(34.1)	255(1.3)		26731(26.9)	276(1.0)	
Occasionally	28484(50.2)	296(1.0)		56336(56.6)	503(0.9)	
Never	1546(2.7)	44(2.8)		3631(3.6)	63(1.7)	
Eggs			<0.0001			<0.0001
Daily	2931(5.2)	56(1.9)		3475(3.5)	60(1.7)	
Weekly	20682(36.5)	317(1.5)		28778(28.9)	363(1.3)	
Occasionally	19786(34.9)	201(1.0)		32635(32.8)	287(0.9)	
Never	13330(23.5)	146(1.1)		34647(34.8)	340(1.0)	
Fish			<0.0001			<0.0001
Daily	3706(6.5)	90(2.4)		6505(6.5)	149(2.3)	
Weekly	14414(25.4)	238(1.7)		22070(22.2)	304(1.4)	
Occasionally	21818(38.5)	225(1.0)		34242(34.4)	264(0.8)	
Never	16782(29.6)	167(1.0)		36724(36.9)	331(0.9)	
Chicken or meat			<0.0001			<0.0001
Daily	706(1.2)	6(0.9)		839(0.8)	14(1.7)	
Weekly	15609(27.5)	269(1.7)		21938(22.0)	292(1.3)	
Occasionally	26135(46.1)	291(1.1)		42222(42.0)	423(1.0)	
Never	14272(25.2)	155(1.1)		34537(34.7)	320(0.9)	
Body Mass Index (kg/m^2^)			<0.0001			<0.0001
≤18.5 (Underweight)	15358(28.7)	96(0.8)		30663(32.1)	119(0.5)	
18.5-22.9 (Normal)	26616(49.8)	288(1.0)		41219(43.2)	319(0.7)	
23.0-24.9 (Overweight)	5635(10.5)	128(2.3)		9454(9.9)	153(1.6)	
≥25.0 (Obese)	5881(11.0)	178(3.0)		14169(14.8)	437(3.1)	
Current Tobacco smoking			0.498			0.514
No	35422(62.4)	450(1.3)		97738(98.2)	1030(1.1)	
Yes	21321(37.6)	270(1.3)		1835(1.8)	19(1.0)	
Alcohol consumption			0.362			0.020
Never	35965(63.4)	436(1.2)		97101(97.5)	1037(1.1)	
Occasionally	13054(23.0)	180(1.4)		1067(1.1)	7(0.7)	
Once a week	5676(10.0)	74(1.3)		1010(1.0)	3(0.3)	
Almost everyday	2048(3.6)	31(1.5)		396(0.4)	1(0.3)	
Frequency of watching TV			<0.0001			<0.0001
Not at all	10517(18.5)	112(1.1)		35399(35.6)	255(0.7)	
Less than once a week	11420(20.1)	95(0.8)		10438(10.5)	96(0.9)	
At least once a week	9081(16.0)	114(1.3)		10952(11.0)	100(0.9)	
Almost everyday	25717(45.3)	400(1.6)		42763(43.0)	598(1.4)	
Age			<0.0001			<0.0001
20-29	22842(40.3)	91(0.4)		43196(43.4)	113(0.3)	
30-39	19045(33.6)	179(0.9)		33522(33.7)	342(1.0)	
40-49	14855(26.2)	450(3.0)		22856(23.0)	594(2.6)	
Education a			<0.0001			<0.0001
Illiterate	11607(20.5)	144(1.2)		45113(45.3)	338(0.7)	
Literate, < middle school	10030(17.7)	111(1.1)		14463(14.5)	192(1.3)	
Middle school completed	26783(47.2)	320(1.2)		31665(31.8)	435(1.4)	
High school complete and above	8311(14.7)	146(1.8)		83284(8.4)	83(1.0)	
Religion			0.099			<0.0001
Hindu	46727(82.3)	575(1.2)		80648(81.0)	792(1.0)	
Muslim	6841(12.1)	103(1.5)		12940(13.0)	164(1.3)	
Christian	1290(2.3)	19(1.5)		2526(2.5)	56(2.2)	
Sikhs	1009(1.8)	17(1.7)		1836(1.8)	21(1.1)	
Others b	876(1.5)	6(0.7)		1624(1.6)	16(1.0)	
Caste/tribe c			<0.0001			<0.0001
Scheduled caste	10670(18.8)	131(1.2)		18260(18.3)	173(0.9)	
Scheduled tribes	4732(8.3)	24(0.5)		8002(8.0)	30(0.4)	
Other backward class	22116(39.0)	256(1.2)		38860(39.0)	368(0.9)	
Others	17414(30.7)	270(1.6)		31440(31.6)	437(1.4)	
Missing caste	1810(3.2)	40(2.2)		3011(3.0)	41(1.4)	
Wealth index d			<0.0001			<0.0001
Lowest	9103(16.0)	71(0.8)		17286(17.4)	71(0.4)	
Second	10205(18.0)	100(1.0)		18546(18.6)	141(0.8)	
Middle	11533(20.3)	80(0.7)		19698(19.8)	152(0.8)	
Fourth	12634(22.3)	154(1.2)		20925(21.0)	275(1.3)	
Highest	13266(23.4)	316(2.4)		23119(23.2)	411(1.8)	
Place of residence			<0.0001			<0.0001
Urban	20779(36.6)	347(1.7)		33355(33.5)	551(1.7)	
Rural	35963(63.4)	373(1.0)		66219(66.5)	498(0.8)	
Total percent, Diabetes		1.3			1.1	
Number e	56742	720		99574	1050	

a Education: illiterate (0 years of education), literate but less than middle school complete (1–5 years of education), middle school complete (6–8 years of education), high school complete or more (9+ years of education).

b Others include Buddhist, Jain, Jewish, Zoroastrian.

c Scheduled castes and scheduled tribes are identified by the Government of India as socially and economically backward and needing protection from social injustice and exploitation. Other backward class is a diverse collection of intermediate castes that were considered low in the traditional caste hierarchy but are clearly above scheduled castes. Others is thus a default residual group that enjoys higher status in the caste hierarchy.

d The wealth index is based on following assets in the household: household electrification, type of windows, drinking water source, type of toilet facility, type of flooring, material of exterior walls, type of roofing, house ownership, ownership of a bank or post office account, and ownership of a mattress, a pressure cooker, a chair, a cot/bed, a table, an electric fan, a radio/transistor, a black and white television, a colour television, a sewing machine, a mobile telephone, any other telephone, a computer, a refrigerator, a watch or clock, a bicycle, a motorcycle or scooter, an animal-drawn cart, a car, a water pump, a thresher, and a tractor.

e Number of men and women varies slightly for individual variables depending on the number of missing values.

**Table 2:** Unadjusted and adjusted effect (odds ratios with 95% CI) of frequency of consumption of specific food items and selected factors on the risk of diabetes among men, India, 2005-06.

Predictors and confounders	Men	
	Model 1 Unadjusted OR(95%CI)	Model 2 Adjusted OR(95%CI)
Milk or curd		
Daily	0.82(0.64-1.06)	0.79(0.60-1.06)
Weekly	0.56(0.42-0.75)	0.64(0.47-0.88)
Occasionally	0.52(0.39-0.69)	0.60(0.44-0.81)
Never R	1.00	1.00
Pulses and beans		
Daily	0.57(0.32-0.99)	0.63(0.35-1.14)
Weekly	0.39(0.22-0.69)	0.48(0.27-0.87)
Occasionally	0.42(0.23-0.79)	0.52(0.28-0.99)
Never R	1.00	1.00
Green leafy vegetables		
Daily	1.31(0.93-1.85)	0.99(0.69-1.43)
Weekly	1.18(0.83-1.68)	1.15(0.79-1.66)
Never/Occasionally	1.00	1.00
Fruits		
Daily	0.59(0.42-0.83)	0.33(0.22-0.50)
Weekly	0.45(0.33-0.63)	0.34(0.23-0.49)
Occasionally	0.36(0.26-0.49)	0.39(0.28-0.56)
Never R	1.00	1.00
Eggs		
Daily	1.74(1.28-2.38)	1.40(0.91-2.13)
Weekly	1.40(1.15-1.71)	1.31(0.95-1.81)
Occasionally	0.92(0.74-1.14)	1.07(0.79-1.47)
Never R	1.00	1.00
Fish		
Daily	2.48(1.92-3.22)	2.46(1.66-3.65)
Weekly	1.67(1.37-2.03)	1.77(1.24-2.53)
Occasionally	1.04(0.85-1.27)	1.37(0.97-1.94)
Never R	1.00	1.00
Chicken or meat		
Daily	0.79(0.35-1.79)	0.31(0.12-0.82)
Weekly	1.60(1.31-1.95)	0.96(0.65-1.42)
Occasionally	1.03(0.84-1.25)	0.79(0.55-1.15)
Never R	1.00	1.00
Body Mass Index (kg/m^2^)		
≤18.5 (Underweight)		0.84(0.67-1.06)
18.5-22.9 (Normal) R		1.00
23.0-24.9 (Overweight)		1.56(1.25-1.94)
≥25.0 (Obese)		1.78(1.44-2.20)
Current Tobacco smoking		
No R		1.00
Yes		0.92(0.78-1.09)
Alcohol consumption		
Never R		1.00
Occasionally		1.15(0.94-1.39)
Once a week		0.94(0.71-1.23)
Almost everyday		0.97(0.65-1.44)
Frequency of watching TV		
Not at all R		1.00
Less than once a week		0.90(0.67-1.19)
At least once a week		1.24(0.93-1.65)
Almost everyday		0.92(0.70-1.22)
Age		
20-29^R^		1.00
30-39		2.16(1.66-2.82)
40-49		7.19(5.65-9.16)
Education		
Illiterate R		1.00
Literate, <middle school		0.78(0.60-1.01)
Middle school completed		0.73(0.57-0.94)
High school complete and above		0.73(0.53-0.99)
Religion		
Hindu R		1.00
Muslim		1.07(0.83-1.38)
Christian		0.70(0.43-1.15)
Sikhs		0.91(0.54-1.52)
Others		0.42(0.17-1.04)
Caste/tribe		
Scheduled caste R		1.00
Scheduled tribes		0.45(0.29-0.70)
Other backward class		0.78(0.62-0.97)
Others		0.78(0.62-0.99)
Missing caste		1.28(0.84-1.94)
Wealth index		
Lowest R		1.00
Second		1.36(0.99-1.88)
Middle		0.89(0.62-1.27)
Fourth		1.60(1.12-2.28)
Highest		2.98(2.01-1.41)
Place of residence		
Urban		1.07(0.89-1.29)
Rural R		1.00

For variable definition see Table 1; ^R^ Reference category; Model 1 unadjusted; Model 2 adjusted for all

**Table 3:** Unadjusted and adjusted effect (odds ratios with 95% CI) of frequency of consumption of specific food items and selected factors on the risk of diabetes among women, India, 2005-06.

Predictors and confounders	Women	
	Model 1 Unadjusted OR(95%CI)	Model 2 Adjusted OR(95%CI)
Milk or curd		
Daily	1.17(0.96-1.44)	1.06(0.85-1.33)
Weekly	0.88(0.68-1.12)	0.94(0.73-1.23)
Occasionally	0.88(0.71-1.09)	0.99(0.79-1.24)
Never R	1.00	1.00
Pulses and beans		
Daily	0.43(0.27-0.67)	0.51(0.32-0.81)
Weekly	0.41(0.26-0.65)	0.51(0.32-0.81)
Occasionally	0.57(0.36-0.92)	0.68(0.42-1.10)
Never R	1.00	1.00
Green leafy vegetables		
Daily	0.80(0.64-1.01)	0.96(0.75-1.23)
Weekly	0.76(0.60-0.97)	1.03(0.80-1.33)
Never/Occasionally	1.00	1.00
Fruits		
Daily	0.93(0.70-1.24)	0.44(0.32-0.61)
Weekly	0.59(0.45-0.78)	0.36(0.27-0.49)
Occasionally	0.51(0.39-0.67)	0.46(0.34-0.61)
Never R	1.00	1.00
Eggs		
Daily	1.76(1.34-2.33)	1.00(0.70-1.43)
Weekly	1.29(1.11-1.50)	0.99(0.77-1.27)
Occasionally	0.51(0.39-0.67)	0.94(0.74-1.20)
Never R	1.00	1.00
Fish		
Daily	2.58(2.13-3.14)	1.72(1.26-2.33)
Weekly	1.54(1.32-1.80)	1.41(1.07-1.87)
Occasionally	0.86(0.73-1.01)	0.94(0.71-1.25)
Never R	1.00	1.00
Chicken or meat		
Daily	1.84(1.08-3.15)	1.02(0.53-1.98)
Weekly	1.44(1.23-1.69)	1.05(0.77-1.44)
Occasionally	1.08(0.94-1.25)	1.21(0.90-1.61)
Never R	1.00	1.00
Body Mass Index (kg/m^2^)		
≤18.5 (Underweight)		0.69(0.56-0.85)
18.5-22.9 (Normal) R		1.00
23.0-24.9 (Overweight)		1.59(1.30-1.94)
≥25.0 (Obese)		2.37(2.01-2.79)
Current Tobacco smoking		
No R		1.00
Yes		1.24(0.77-2.00)
Alcohol consumption		
Never R		1.00
Occasionally		0.86(0.40-1.83)
Once a week		0.51(0.15-1.68)
Almost everyday		0.63(0.12-3.37)
Frequency of watching TV		
Not at all R		1.00
Less than once a week		0.96(0.75-1.22)
At least once a week		0.80(0.62-1.03)
Almost everyday		0.91(0.75-1.11)
Age		
20-29^R^		1.00
30-39		3.31(2.66-4.12)
40-49		8.03(6.49-9.93)
Education		
Illiterate R		1.00
Literate, <middle school		1.38(1.14-1.68)
Middle school completed		1.49(1.24-1.79)
High school complete and above		0.99(0.73-1.34)
Religion		
Hindu R		1.00
Muslim		1.15(0.94-1.40)
Christian		1.51(1.12-2.03)
Sikhs		0.83(0.53-1.30)
Others		0.91(0.54-1.55)
Caste/tribe		
Scheduled caste R		1.00
Scheduled tribes		0.51(0.33-0.77)
Other backward class		0.91(0.75-1.11)
Others		1.00(0.82-1.22)
Missing caste		0.91(0.62-1.32)
Wealth index		
Lowest R		1.00
Second		1.58(1.17-2.12)
Middle		1.30(0.96-1.77)
Fourth		1.66(1.21-2.27)
Highest		1.62(1.14-2.30)
Place of residence		
Urban		1.44(1.24-1.68)
Rural R		1.00

For variable definition see Table 1; ^R^ Reference category; Model 1 unadjusted; Model 2 adjusted for all
